# Supplementary material for: Effect of Paclobutrazol Application on Enhancing the Efficacy of Nitenpyram against the Brown Planthopper, Nilaparvata lugens
Source: Int J Mol Sci. 2023 Jun 22;24(13):10490. doi: 10.3390/ijms241310490 (PMC10341613; doi:10.3390/ijms241310490)
Supplement: Supplementary file 1 [file ijms-24-10490-s001.zip › ijms-2402452-supplementary.pdf]

Supporting Information

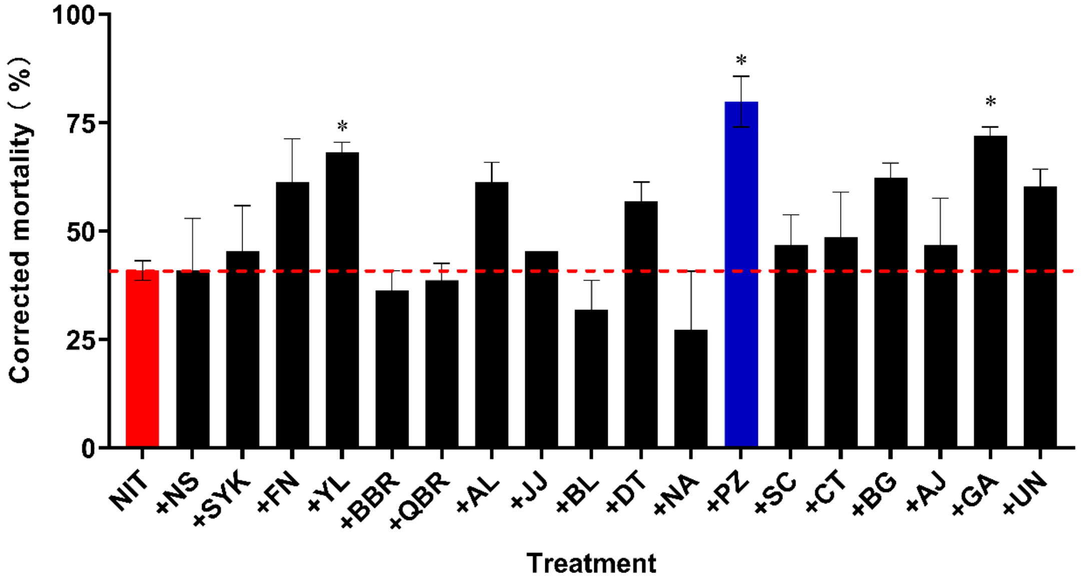

Figure S1. Toxicity of the chemical mixtures against BPH nymphs. The bioassay was carried out on 7th day after the host plants were dipped and the mortalities were recorded after 96 h. Data are presented as the means ± S.E. for three independent replicates. The red dashed line represents the average corrected mortality of NIT treatment. The bars with asterisk indicate significant differences between the treatments of NIT and chemical mixtures (*p* <0.05, Tukey's multiple range test).

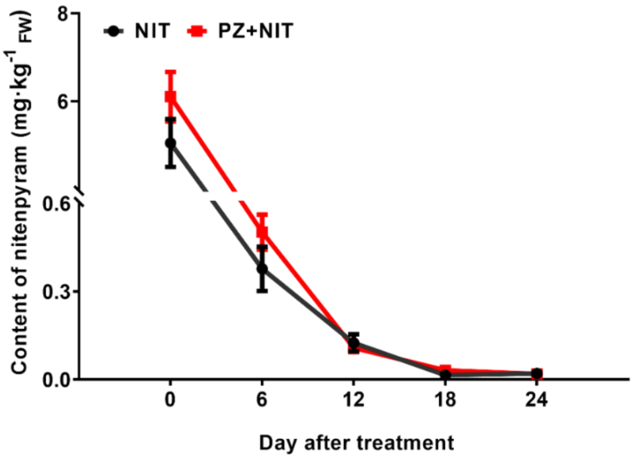

Figure S2. Variation of nitenpyram content in rice stem after exposure to insecticide within a period of 24-day. Data are presented as the means ± S.E. for four independent replicates (Student's *t*-test).
